# RETRACTION: Ear Keloids: An Innovative 3‐Steps Combined Treatment

**DOI:** 10.1111/srt.70328

**Published:** 2026-01-19

**RETRACTION**: D. Piccolo, G. Crisman, D. Bollero, B. Bovani, A. Gennai, F. Melfa, M. Tretti Clementoni, and C. Conforti, “Ear Keloids: An Innovative 3‐Steps Combined Treatment,” *Skin Research and Technology* 29, no. 11 (2023): e13506, https://doi.org/10.1111/srt.13506.

The above article, published online on 27 October 2023 in Wiley Online Library (wileyonlinelibrary.com), has been retracted by agreement between Skin Research and Technology; and John Wiley & Sons Ltd. The article was accepted for publication following an insufficient peer review process that does not meet the standards of the journal's peer review policy. Furthermore, several inconsistencies between figures and relative legends were identified, and the case presented in Figure 2 appears to have been previously reported in a separate publication by the same author group, despite the articles describing different treatments and study designs. Accordingly, the article has been retracted. The authors have been informed of the decision of retraction but unavailable for final confirmation.

